# Antigenicity and immunogenicity of different morphological forms of *Borrelia burgdorferi* sensu lato spirochetes

**DOI:** 10.1038/s41598-024-54505-y

**Published:** 2024-02-18

**Authors:** Kristyna Sloupenska, Barbora Koubkova, Pavel Horak, Jana Dolezilkova, Beata Hutyrova, Mojmir Racansky, Martina Miklusova, Jan Mares, Milan Raska, Michal Krupka

**Affiliations:** 1 Department of Immunology, Faculty of Medicine and Dentistry, Palacky University Olomouc, Hnevotinska 3, 779 00 Olomouc, Czech Republic; 2 Department of Allergology and Clinical Immunology, University Hospital Olomouc, Zdravotniku 248/7, 779 00 Olomouc, Czech Republic; 3 Third Department of Internal Medicine-Nephrology, Rheumatology and Endocrinology, University Hospital Olomouc, Zdravotniku 248/7, 779 00 Olomouc, Czech Republic; 4 Laboratory of Medical Parasitology and Zoology, Public Health Institute Ostrava, Partyzanske Namesti 2633/7, Moravska Ostrava, 702 00 Ostrava, Czech Republic; 5 Department of Neurology, University Hospital Olomouc, Zdravotniku 248/7, 779 00 Olomouc, Czech Republic; 6 Department of Neurology, Faculty of Medicine and Dentistry, Palacky University Olomouc, Hnevotinska 3, 779 00 Olomouc, Czech Republic; 7 Department of Immunology, University Hospital Olomouc, Zdravotniku 248/7, 779 00 Olomouc, Czech Republic; 8 Third Department of Internal Medicine-Nephrology, Rheumatology and Endocrinology, Faculty of Medicine and Dentistry, Palacky University Olomouc, Hnevotinska 3, 779 00, Olomouc, Czech Republic

**Keywords:** Biochemistry, Immunology, Microbiology

## Abstract

*Borrelia burgdorferi* sensu lato is a species complex of pleomorphic spirochetes, including species that cause Lyme disease (LD) in humans. In addition to classic spiral forms, these bacteria are capable of creating morphological forms referred to as round bodies and aggregates. The subject of discussion is their possible contribution to the persistence of infection or post-infection symptoms in LD. This study investigates the immunological properties of these forms by monitoring reactivity with early (n = 30) and late stage (n = 30) LD patient sera and evaluating the immune response induced by vaccination of mice. In patient sera, we found a quantitative difference in reactivity with individual morphotypes, when aggregates were recognized most intensively, but the difference was statistically significant in only half of the tested strains. In post-vaccination mouse sera, we observed a statistically significant higher reactivity with antigens p83 and p25 (OspC) in mice vaccinated with aggregates compared to mice vaccinated with spiral forms. The importance of the particulate nature of the antigen for the induction of a Th1-directed response has also been demonstrated. In any of morphological forms, the possibility of inducing antibodies cross-reacting with human nuclear and myositis specific/associated autoantigens was not confirmed by vaccination of mice.

## Introduction

*Borrelia burgdorferi* sensu lato (Bbsl) is a species group of spirochetal bacteria containing at least nine species with the potential to cause Lyme disease (LD) in humans. Most human infections are caused by 3 genospecies: *B. afzelii*, *B. garinii*, and *B. burgdorfer*i sensu stricto^1–3^. The infection has the character of an anthropozoonosis, the vector is ticks of the genus *Ixodes*^4^. It is a multisystemic disease with variable and often non-specific symptoms^1,5–8^.

Although antibiotic treatment of LD is effective in most cases, in some patients, chronic problems persist even after correctly indicated therapy. The term post-treatment Lyme disease syndrome (PTLDS) has been proposed for these conditions^7,9,10^. The cause of this condition is not yet known, among the discussed possibilities are post-infection autoimmune reactions, persistence of some forms of bacteria or their immunogenic parts or the influence of co-infections carried by ticks^10–14^.

Bbsl are variable in many ways. *Borrelia* must be able to survive in the organism of the tick, transfer to the organism of the vertebrate host, escape its immune system and disseminate to the target tissue. Adaptation to such different conditions is made possible by a regulated change in the expression of surface lipoproteins based on environmental signals such a change in temperature or pH during blood sucking by the tick. During the transition of *Borrelia* to the mammalian organism, the expression of proteins such as Outer surface proteins A and B (OspA, OspB) necessary for survival in the tick is suppressed and the expression of proteins important for survival in the mammalian organism, such as Outer surface protein C (OspC) or Decorin binging proteins A and B (DbpA, DbpB) is triggered^15–17^. In vitro, the expression of these proteins changes depending on the culture conditions, including temperature^18^. Another lipoprotein, the 35-kDa surface-exposed VlsE (Vmp-like sequence, expressed) is essential for escaping mammalian immunity due to its genetic recombination-induced antigenic variability, which takes place only in vivo, during infection. Antibodies against the invariant region of this protein are important for the serological diagnosis of LD^19–21^. The aforementioned Osp antigens are also highly variable in sequence between as well as within individual *Borrelia* species, which is one of the main reasons for the absence of an available vaccine against LD^22^.

Under optimal living conditions, *Borrelia* have a classic spiral form, but under the influence of adverse factors such as osmotic shock, lack of nutrients or the presence of certain antibiotics, they can change into spherical forms. These forms do not yet have a fixed nomenclature, they are most often called cysts or round bodies. However, unlike classic bacterial cysts, they do not form a thick cell wall, instead they are formed by the coiling of a protoplasmic cylinder into a pouch formed by an outer membrane^23–27^. Several works describe the possibility of reversion of round bodies back into the spiral forms in vitro^27,28^ as well as in vivo, in experimental mice^29^, but the significance of this phenomenon in the pathogenesis of LD and/or PTLDS remains unclear and it is the subject of discussion^11,30,31^.

Another morphological variant is aggregates, sometimes referred to as biofilm or biofilm-like structures, which are formed by clusters of bacteria with a predominantly spiral morphology. These were observed in vitro in aged cultures but also in midgut of ticks^32,33^. More detailed information on the mechanism of formation and biological significance of aggregates, similarly to round bodies, is still missing.

Although little information is so far available on the importance of alternative morphological forms of *B. burgdorferi* in the pathogenesis of LD, there are emerging opinions about the suitability of their inclusion in diagnostic tools^34,35^. In order to prove the legitimacy of this proposal, additional experimental data focused mainly on the immunological properties of the mentioned forms will be necessary.

In our study, we focused on testing the antigenicity of different in vitro induced morphological forms of 4 species of the Bbsl. complex (*B. burgdorferi* s.s., *B. afzelii*, *B. garinii* a *B. bissettii*) using a reaction with the sera of LD patients with bacterial lysates in ELISA format. Immunogenicity of individual *Borrelia* morphotypes was then tested in experimental mice immunized with inactivated morphotypes of *B. garinii*.

## Results

### Reactivity of LD patient sera with individual morphological forms of *Borrelia* sp.

**Figure 1:**
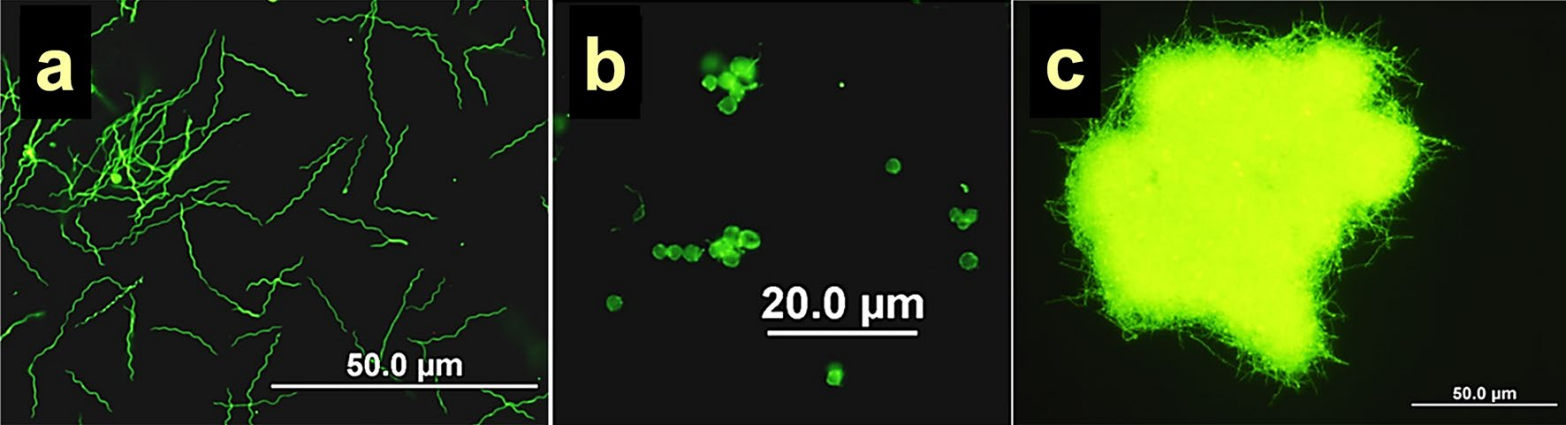
Morphological forms of *Borrelia burgdorferi* s. l. bacteria. Figure shows Carboxyfluorescein succinimidyl ester staining of *B. garini* strain. (**a**) Spiral “classic" form, (**b**) round bodies, (**c**) aggregate.

First we compared the reactivity of sera of patients stratified based on serological markers of the early and late phase of LD with lysates of four *Borrelia* genospecies induced to form aggregates, round bodies or spiral forms. The change in morphology was confirmed by fluorescence microscopy before each experiment (Fig. 1). We used the ELISA panels coated with lysates of individual morphological forms from *B. burgdorferi* s.s., *B. afzelii*, *B. garinii* and *B. bissettii*.

**Figure 2:**
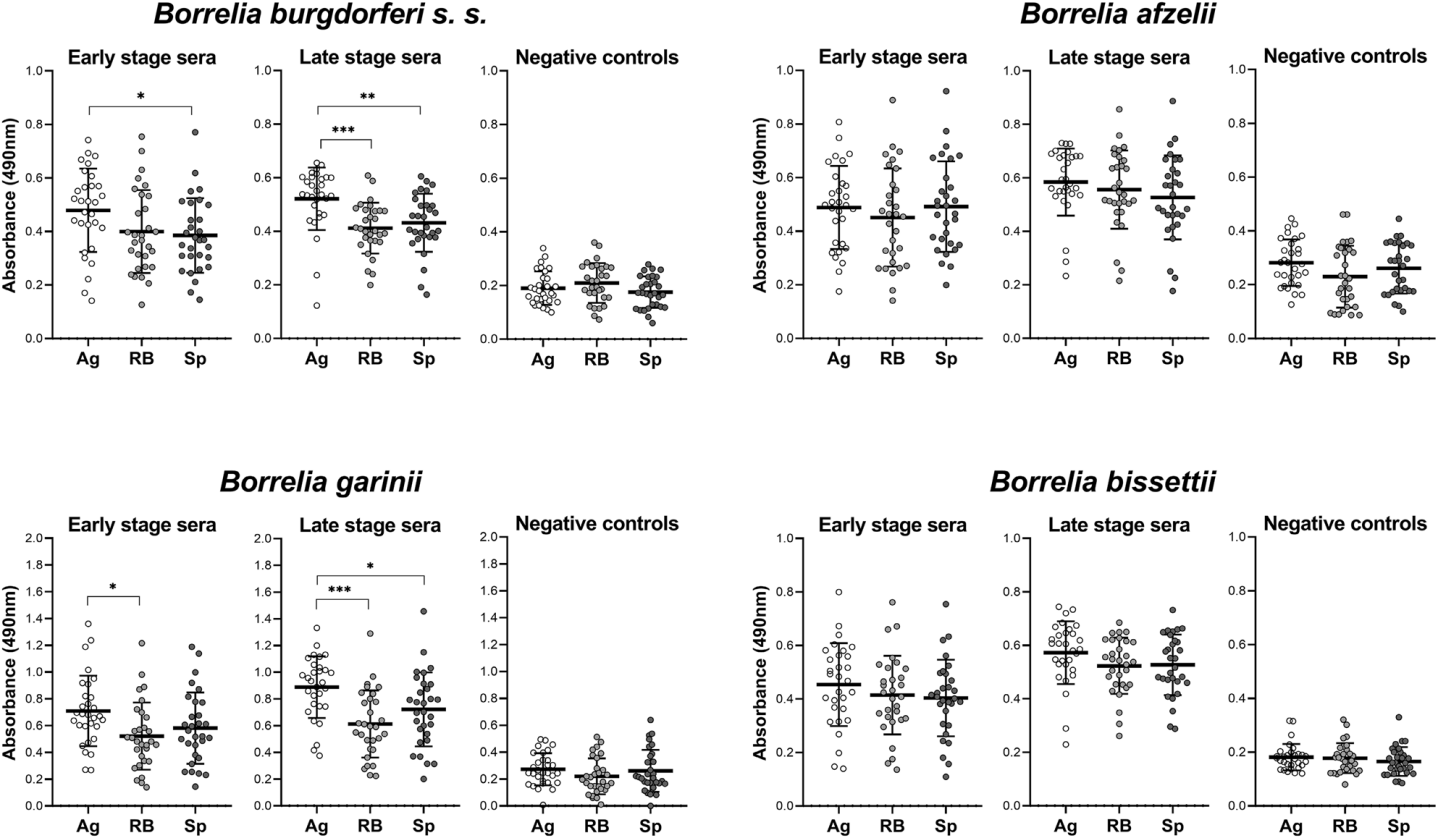
Reactivity of LD patients’ sera positive for markers of early and late stage of disease. The sera of subjects seropositive for markers of early stage of LD (n = 30), late stage of LD (n = 30) and seronegative control subjects (n = 30) were tested. Borrelia-specific IgG antibodies were determined by the ELISA method with panels coated with lysates of individual morphotypes. Results are shown as absorbance of individual wells at a wavelength of 490 nm at a 100× sample dilution. The graphs show a comparison of the tested groups reactivity with the individual morphotypes in order to illustrate the differences in their antigenicity. *Ag *aggregates, *RB* round bodies, *Sp* spiral forms, *p < 0.05, **p < 0.01, ***p < 0.001.

We analyzed whether the sera of early stage LD patients differ from late stage LD patients in the proportional recognition of aggregates, round bodies, and spiral forms for individual *Borrelia* genospecies. The highest reaction was recorded for aggregates from all species, but, due to the high interindividual variability, the statistical significance was recorded only for two species—*B. burgdorferi* s.s. and *B. garinii* (Fig. 2). For both species, the sera of late stage patients reacted stronger with aggregates than with round bodies and spiral forms. In early stage patients cohort, the differences were less prominent with *B. burgdodrferi* s.s. Statistically significant differences were not found for *B. bissettii* and *B. afzelii*. When comparing individual groups, the reactions of both early and late patient sera were statistically significantly stronger than negative control sera (Supplementary Fig. S1). The average level of specific antibodies was in all cases higher in late phase than in early phase patient sera, but due to the variability of sera reactivity within the groups, the difference achieved statistical significance only in following groups: in *Borrelia* aggregates , the difference was statistically significant for *B. afzelii* (p < 0.05), *B. garinii* (p < 0.01) and *B. bissettii* (p < 0.001), in round bodies for *B. afzelii* (p < 0.05) and *B. bissettii* (p < 0.001). In the case of spiral forms only in *B. bissettii* lysates were differently recognized by the sera (p < 0.001).

**Figure 3:**
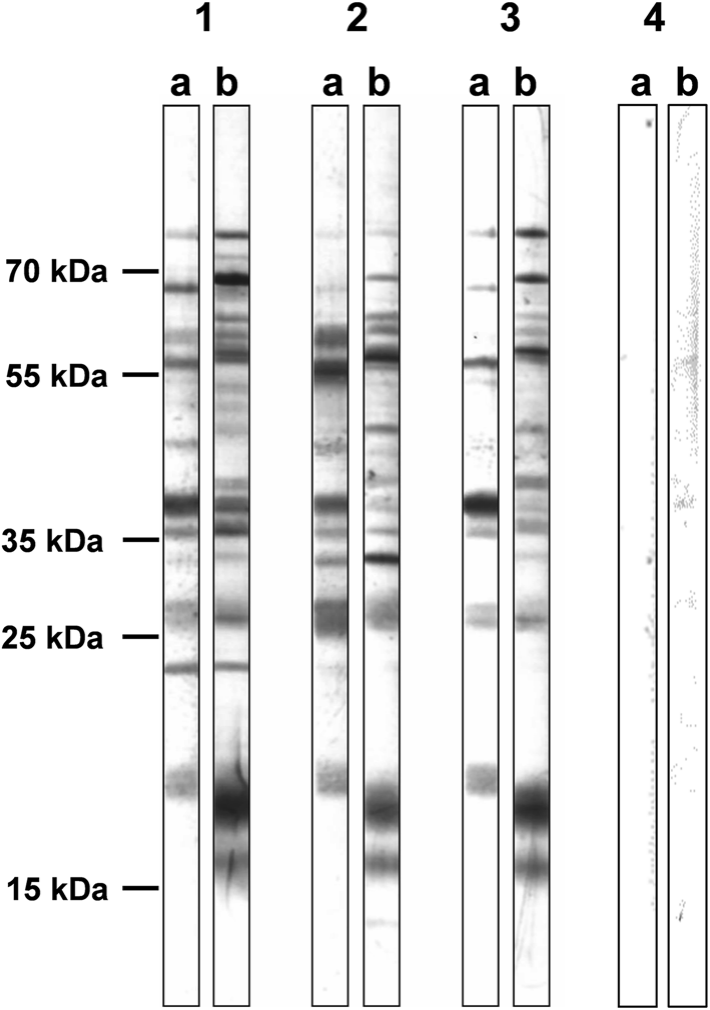
Immunoblot analysis of the reactivity of patients sera with lysates of spiral forms and aggregates of *B. garinii*. 1, 2, 3—Individual positive sera, 4—negative control serum, (**a**) Lysate of spiral forms, (**b**) lysate of aggregates.

To specify observed differences in LD patient’s sera reactivities with individual antigens of *Borrelia* spirals and aggregates (Fig. 2), three selected sera were reacted with SDS-PAGE-separated lysates of *B. garinii* spiral forms and aggregates using immunoblot method (Fig. 3**,** uncropped blots can be found as Supplementary Fig. S2)*.* A similar pattern is evident in all three samples, with reactive antigen in the region above 15 kDa in the aggregates, which is not present or recognized in the lysate from spiral forms, and antigen in the region around 20 kDa present in both forms but reacting more strongly in the aggregates. Two antigens below and above the 70 kDa region also react more strongly with aggregates. On the contrary, a stronger band in the area above 35 kDa is evident in the spiral form lysate.

### The immunogenicity of dominant *Borrelia* antigens presented in spirals, round bodies, and aggregates

Next we prepared inactivated whole-cell vaccines from in vitro produced spirals, round bodies, and aggregates of *B. garini*. Vaccines were used for intradermal immunization of experimental mice and hyperimmune sera reactivities with various *Borrelia* antigens were analyzed by ELISA and by routine diagnostic western blot and line blot strips (EUROLINE).

Routine in vitro diagnostic *Borrelia* blot kits were adapted by replacing the secondary anti-human IgG antibody with anti-mouse IgG antibody (both AP conjugated). This allowed us to identify and densitometrically quantify the reaction of mouse sera with a number of diagnostically significant *Borrelia* antigens.

**Figure 4:**
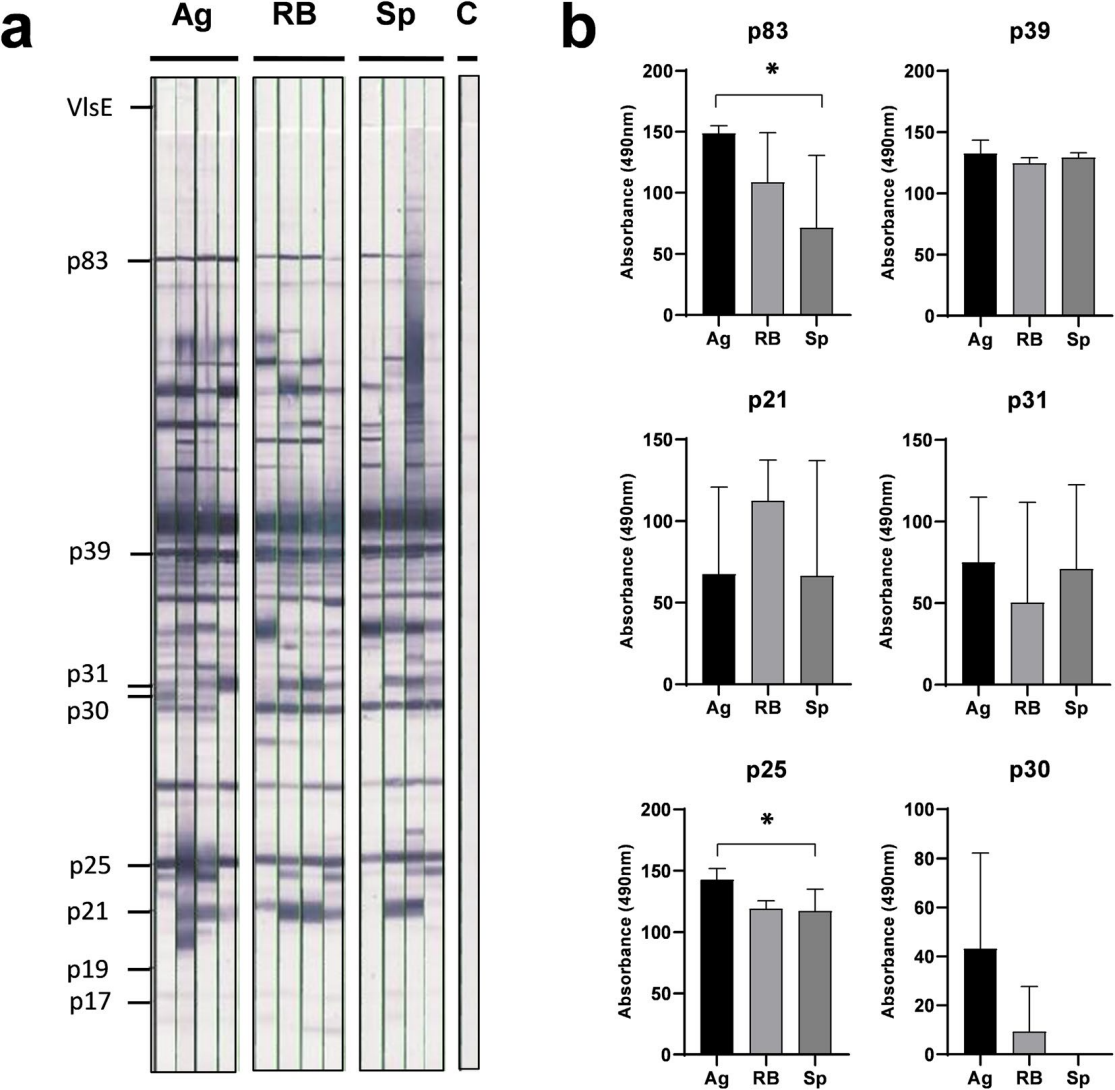
Western blot analysis of sera of mice immunized with inactivated whole cell vaccines generated from individual morphological types of *B. garinii*. The results were evaluated by EUROLineScan software enabling the identification and densitometric quantification of the reaction with specific antigens. (**a**) Blot strips incubated with sera from individual vaccinated mice. (**b**) Densitometric evaluation of reactivity in individual groups for antigens recognized by the software, with the exception of antigens p17 and p19, where a negative reaction was recorded in all mice. *Ag* aggregates, *RB* round bodies, *Sp* spiral forms, *p < 0.05.

The first set of analyses was performed on the classic western blot strips based on SDS-PAGE-separated and western blotted protein extract of *Borrelia* lysate and thus containing entire wide spectrum of *Borrelia* antigens. The results of densitometric analysis of individual bands performed by EUROLineScan software are shown in Fig. 4 (complete software output and example of evaluation can be found as Supplementary Figs. S3 and S4). In all mice, we noticed a uniformly very intense reaction with antigens p39 and p41 (flagellin). Flagellin is not among the antigens evaluated by the used software as diagnostically non-specific, but it is identifiable by the manual evaluation template included in the kit. We noted considerable variability for p21 and p31 (OspA) antigens, where some mice did not respond to these antigens at all. Due to this variability, no significant differences between groups were found for these antigens. High variability was also observed for the p30 antigen, where only one mouse from the group vaccinated with aggregates reacted noticeably. Statistically significant differences were then found for antigens p83 and p25 (OspC) between groups vaccinated with aggregates and spirals (both p < 0.05). None of the mice showed a positive reaction to antigens p17, p19 and, as expected, VlsE as a marker of natural infection. However, the differences between the groups are also evident for several other proteins not identified by the diagnostic software. For an antigen with an approximate molecular weight of 29 kDa, there is a visibly stronger response in the spiral forms- and RB-vaccinated groups than the group vaccinated with aggregates. In the region between 41 and 83 kDa, there are several bands that are positive in the groups vaccinated with aggregates or RB, but absent or only very weak in the group vaccinated with spiral types.

**Figure 5:**
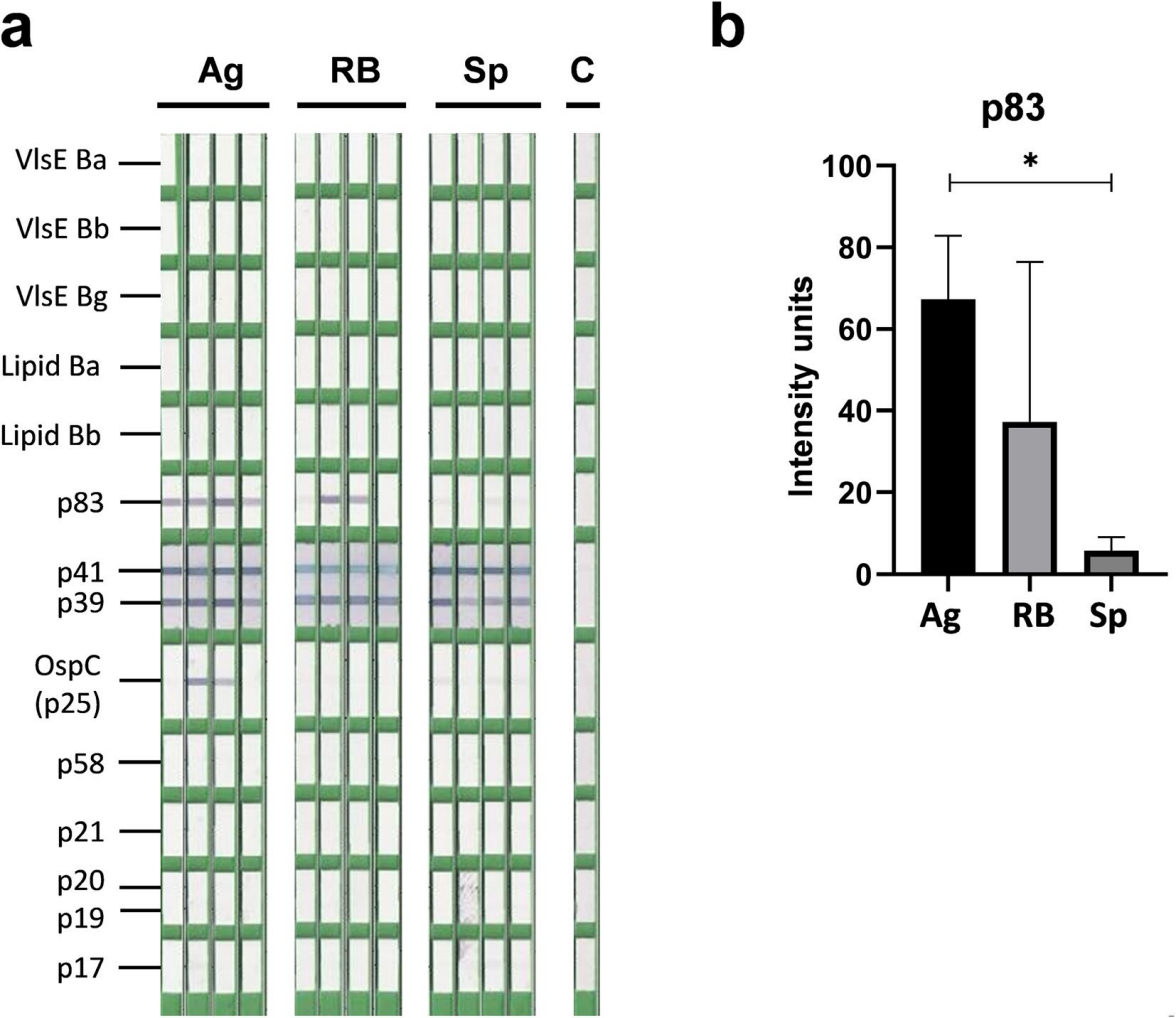
Line blot analysis of sera of mice immunized with inactivated whole cell vaccines generated from individual morphological types of *B. garinii*. The results were evaluated by EUROLineScan software. (**a**) Blot strips incubated with sera from individual vaccinated mice. (**b**) Densitometric evaluation of reactivity in individual groups for antigen p83. *Ag* aggregates, *RB* round bodies, *Sp* spiral forms, *p < 0.05.

The second set of analyses was performed with a line-blot strips containing chips with individual antigens generated by recombinant technology. Here, the reaction with the p39 and p41 antigens was dominant, while with the second mentioned antigen, there was also an oversaturation of the reaction in some sera. For the OspC antigen, a positive reaction was recorded only in two sera from the group vaccinated with *Borrelia* aggregates (Fig. 5a, complete software output can be found as Supplementary Fig. S5), the other two sera from this group and all sera from the other animals were negative. The strongest OspC reaction was noted with the serum analogously to results on western blot strips (Fig. 4). A similar observation was made with the p83 antigen. Only a weak result below the positivity threshold was recorded for two sera of the RB-vaccinated groups and all spiral-vaccinated sera. The difference between the groups found for this antigen was thus significant as assessed by both techniques method (Fig. 5b). The reactions with antigens p18, p19, p20, p21, p58 and lipid antigens were negative in line-blot test for sera all tested sera. Reactions with markers of natural infection—VlsE were also negative.

**Figure 6:**
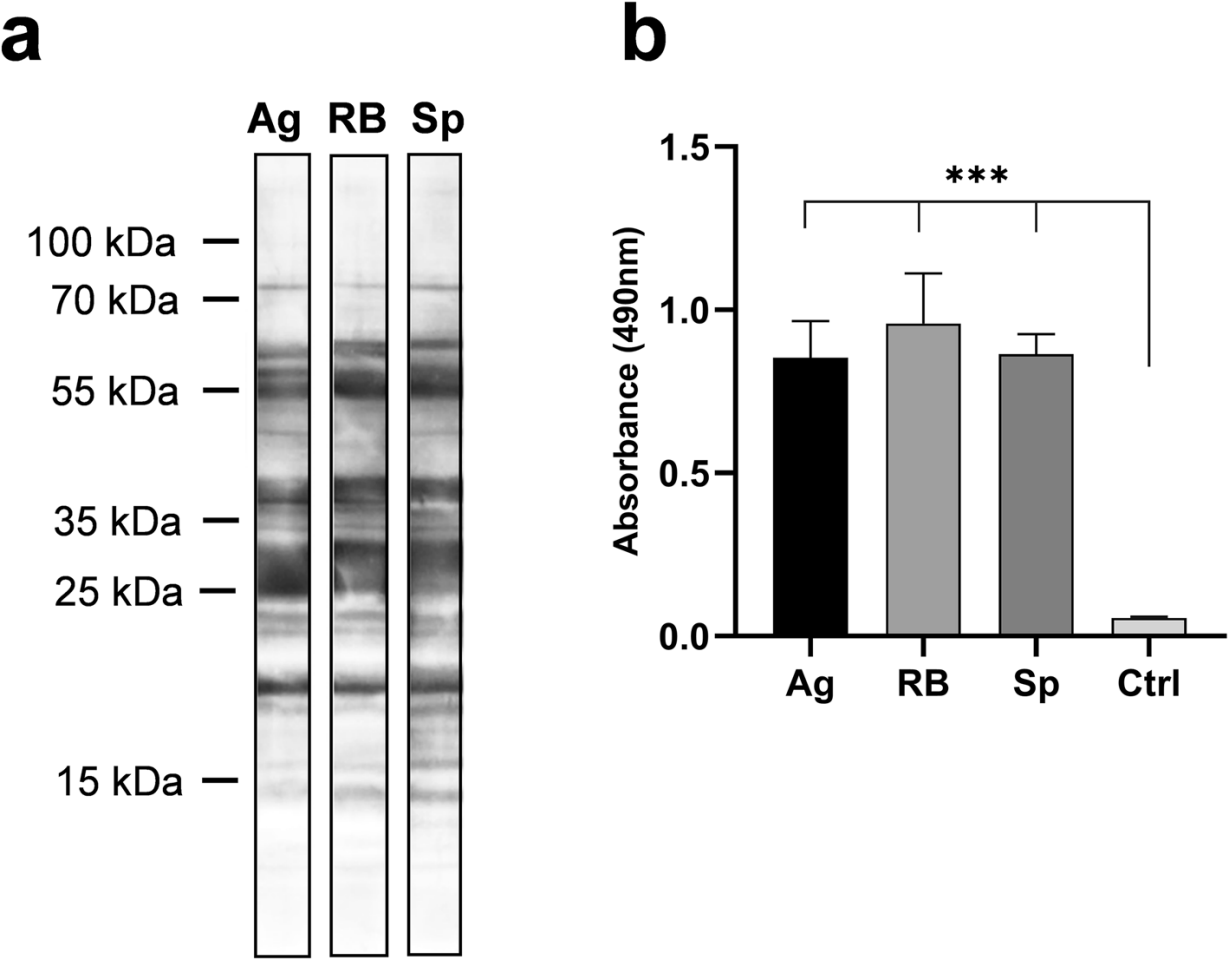
Specific reactivity of serum IgG from mice immunized with inactivated whole cell vaccines generated from individual morphological types of *B. garinii.* Serum IgG reactivity was measured using lysates of *B. garinii* aggregates. (**a**) Lysate of *B. garinii* aggregates were SDS-PAGE separated, western blotted and blot membrane cut into strips. Strips were incubated separately with pooled sera of individual groups of mice. (**b**) Lysate of *B. garinii* aggregates was used for coating on ELISA panel and tested with individual sera. *Ag* sera of mice immunized with aggregates, *RB* mice immunized with round bodies, *Sp* mice immunized with spiral forms, ***p < 0.001.

In order to characterize the reaction of the sera of immunized mice with antigens potentially not expressed in *Borrelia* spiral forms, used for routine western blot tests, we performed analysis testing sera reactivities against the lysate of in vitro induced *B. garinii* aggregates. Using the ELISA platform we tested reactivities of individual sera and using laboratory-made western blot we tested the reactivities of sera pooled separately for aggregate, round bodies and spiral forms-immunized animals (see Methods). The differences in the reactivity of serum pools with individual antigens are visible on the western blot (Fig. 6a**,** uncut blot strips can be found as Supplementary Fig. S6). In contrast, total antigen-specific IgG reactivity determined by the ELISA exhibited no significant differences among individual immunization groups (aggregates, round bodies, spirals, Fig. 6b).

### Comparison of the immunogenicity of inactivated *Borrelia* cells and *Borrelia* lysate-based vaccines toward *Borrelia* spiral form lysate

**Figure 7:**
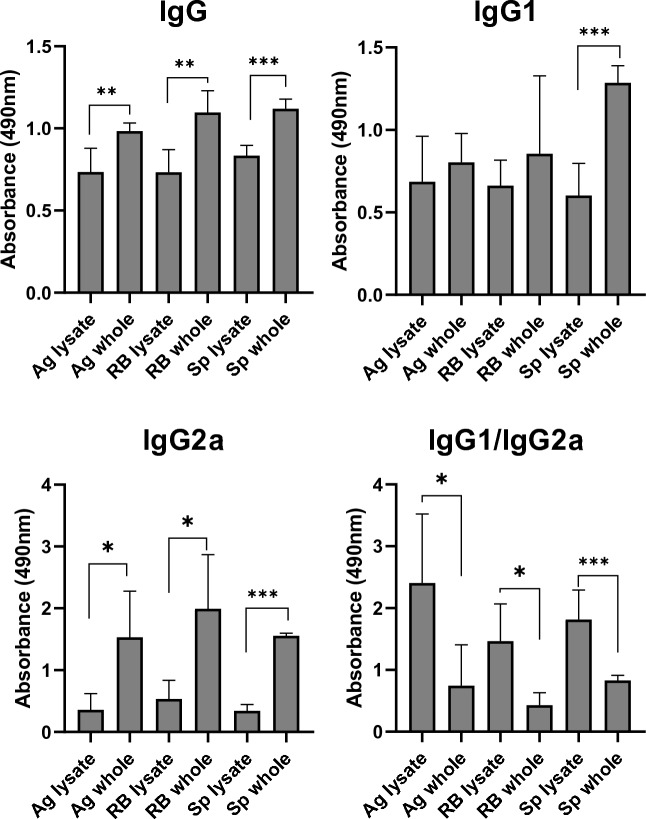
Comparison of the immunogenicity of inactivated whole cell vaccines and cell lysate vaccines generated from spirals, aggregates, and round bodies of *B. garinii* tested in experimental mice. *Borrelia*-specific antibodies were determined by the ELISA method using panels coated equally with lysate of spiral forms of *B. garinii*. *Lysate* lysate vaccine-immunized mice, *whole* inactivated whole cell vaccine-immunized mice, *Ag* sera reactivities of mice immunized with aggregates vaccines, *RB* round bodies, *Sp* spiral forms. *p < 0.05, **p < 0.01, ***p < 0.001.

Finally we performed additional immunization experiment comparing the reactivities of sera from mice after immunization with inactivated whole-cell vaccines, as characterized above, and with lysates of respective *Borrelia* forms (aggregates, round bodies, or spiral forms). We noted higher immunogenicity of inactivated whole-cell vaccines over lysate vaccines (Fig. 7), as determined by the reactivities of total serum IgG with lysate of spiral forms of *B. garinii* in ELISA. Of note, both whole-cell and lysate vaccines doses contained the same amount of total protein (20 µg per one dose) and the same concentration of adjuvant (20% of aluminum adjuvant). The differences in the reactivities of sera from mice immunized with lysate versus whole-cell vaccines made of spiral forms exhibited higher significancy (p < 0.001) than those for aggregates- and round bodies-based vaccines (p < 0.01). In the case of spiral forms, lysate versus whole-cell vaccines significant differences in reactivity were detected also for the Th-2 associated IgG1 subclass. In the case of aggregates and round body vaccines the differences between whole-cell and lysate vaccination were statistically inconclusive mainly due to the higher variability of the results. In the case of the Th1-associated IgG2a subclass, a statistically significant higher response elicited by whole-cell vaccines was recorded for all morphological forms tested. The biggest difference was again for spiral form vaccines.

### Determination of the reactivity of mouse sera with human autoantigens

Further we tested whether sera from mice immunized with *Borrelia* spiral forms, round bodies, or aggregates crossreact with human autoantigens to test the possibilities of mimicry involvement in the autoimmunity hypothesis of PTLDS. The possibility of induction of cross-reacting antibodies between *Borrelia* antigens and human autoantigens was tested with two blot sets covering a wide spectrum of organ-nonspecific autoantibodies involved in systemic autoimmune diseases. No borderline or positive result was determined for any of the tested autoantigens in any of the tested groups of mice (Supplementary Fig. S7).

## Discussion

Although descriptions of “clumps” (aggregates), “blebs” and “gemmae” (round bodies) as morphological forms of Bbsl already appear in the early publications describing the causative agents of LD^36,37^, there are still more unanswered questions than answers about the mechanism of their formation and possible involvement in the pathogenesis LD and PTLDS. Gradually, a number of works were devoted to the emergence of morphological forms and a whole range of factors causing the emergence of morphological variants were described *in-vitro*, such as osmotic shock, change in pH or temperature, oxidative stress, cultivation in serum-free medium, human or animal serum, cerebrospinal fluid or the presence of some antibiotics and aging of culture^24,25,28,31,34,38–40^.

The structure of aggregates was studied in more detail by Sapi and colleagues. Using histochemical staining techniques, they described the presence of an extracellular substance containing alginate exopolysaccharides, DNA and calcium ions in the structure of the aggregates, which is typical of bacterial biofilms. However, genes homologous to those encoding proteins of the metabolic pathway for alginate synthesis described in other bacteria were not found in the genome of *Borrelia*^41,42^. The formation of biofilms made up of bacterial cells and extracellular polysaccharide matrix is a known mechanism enabling some pathogens to escape from the immune system and antibiotics, but it can also contribute to tissue damage through inefficient activation of immune mechanisms^43^.

Feng et al. described the potential of stationary phase culture aggregates to cause more severe and more persistent infection than log phase spiral forms in a mouse model^44^. Several works reported the finding of structures corresponding to morphological variants of *Borrelia* in the tissue of infected patients^31,45–48^, but the methodology of the studies does not allow determining their viability. However, both the methodology of the works and the conclusions about the possible connection of such *Borrelia* morphotypes with PTLDS or persistent infection have been criticized in several review papers^30,49^.

Therefore, it is still not entirely clear whether, or in which cases, these forms are part of the *Borrelia* life cycle or whether they are degenerative forms induced by cellular damage caused by external conditions, or whether and how they are involved in human infection or post-infection complications.

Very little information is also available on the immunological properties of individual Bbsl morphotypes, such as immunogenicity and antigenicity . One of the few works on this topic compared spiral forms with round bodies induced by distilled water and described the differences in phagocytosis and processing of these two *Borrelia* forms and distinct cytokine and chemokine production by affected macrophages^35^. The reactivity of these two forms with the sera of seven patients serologically positive for LB was also monitored using the immunoblot method, with the finding of several antigens that reacted with higher intensity in the case of RB. Among these antigens, the authors mentioned proteins with a molecular weight of 39, 60 and 66 kDa, which, based on their molecular weight, were assume to be p39 (BmpA), heat-shock protein GroEL and p66 antigen. The p21 antigen also showed increased reactivity in RB, but only with one positive patient's serum. Al-Robaiy et al. dealed with the antigenicity of RB rather marginally^50^. Their study using canine immune serum, on the other hand, showed reduced immune reactivity of *Borrelia* after the transition to a spherical morphology with a further decrease over time.

In our work, we focused on determining the differences in the antigenicity of spiral forms, RB and aggregates of four species of the Bbsl complex by evaluating their reactivity with the sera of LD patients. Furthermore, we determined the differences in the immunogenicity of these morphological forms generated from *B. garinii* on model immunization of experimental BALB/c mice.

In order to detect possible changes arising during the course of the infection, we tested a group of patients with an acutely diagnosed infection (early stage infection, persistent IgM serological positivity (n = 30)) and a group of patients followed after the late stage of the infection (serologically characterized by the positivity of markers of advanced infection p58, p83 or lipid antigens (n = 30)). The third group consisted of seronegative patients undergoing differential diagnosis for neurological problems (n = 30). For all forms and species of *Borrelia,* serological reactivity with bacterial lysates was significantly higher in both patient groups (Supplementary Fig. 1). However, the reactivity of some negative sera was also quite high. This phenomenon is most likely due to reactivity with non-specific, cross-reacting antigens, mainly flagellin (p41). In previous studies, we have recorded positivity of varying degrees for flagellin in up to 70% of patients examined for neurological problems (unpublished data) using immunoblot method, while the positivity of p41 itself does not affect the overall test result. Among the patient groups, a higher level of specific antibodies was observed in the late stage over early stage group: for *Borrelia* aggregates, statistical significance was achieved in three out of four *Borrelia* strains, for RB in two strains, but for spiral forms only for *B. bissettii* antigens the difference in recognition of early and late stage was significant. Another view of the same data is shown in Fig. 2 where the reactivities of individual sera within patient’s groups are displayed. In this comparison, the higher reactivity with antigens of *Borrelia* aggregates was detected particularly in late stage patient’s sera, although the statistical significance was achieved only in two out of four tested *Borrelia* strains, mainly due to high variability of individual sera reactivities. These results suggest a possible dominance of "late" antigens in morphological variants (particularly aggregates). Therefore, we selected sera from three patients, for analysis of their reactivities with antigens in lysates of *Borrelia* spirals and aggregates using the western blot method (Fig. 3). Marked differences were detected among patients and among the recognized antigens in spiral forms and aggregates however, their identification and verification of the result on a wider range of samples will require further experimental work with the use of additional analytical methods.

To evaluate the immunogenicity and antigenic recognition of individual morphological variants, we generated inactivated whole cells vaccines from individual morphological forms and compared immune response in experimental mice. We evaluated the spectrum of elicited antigen-specific serum antibodies using commercial human LD diagnostic kits modified for use with mouse sera (Fig. 4). In contrast to the publication of Al-Robayi et al.^50^ , we did not find significant difference in the reactivity with the p39 antigen, where we recorded uniformly high reactivity in all immunized mice. On the other hand, in the case of antigen p21, in accordance with this work, we recorded a higher average reactivity in the group vaccinated with RB, but due to the variability between vaccinated mice, the difference was not statistically significant. A significant difference was found between aggregates and spiral forms for antigens p25 (OspC) and p83, indicating partial dominance of these antigens in *Borrelia* aggregates. A corresponding result was also obtained using a kit based on recombinant antigens (Fig. 5). There is also a statistically significant difference in reactivity with p83 between sera of mice vaccinated with aggregates and spirals with intermediate reactivity in RB. All sera then had a negative reaction with p21, which in the previous test had a positive reaction in most sera. Although the effect of the sequence or posttranslational modification-caused variants of this antigen used for western blot and line blot cannot be ruled out, a more likely explanation is the lower sensitivity of tests based on recombinant antigens. Both tests had a completely negative reaction with all variants of the VlsE antigen, which is the main IgG serological marker in clinical diagnosis. Expression of this protein is typical for natural infection but VlsE expression was confirmed also in vitro^51^. Negative result detected in our analyses (Figs. 4 and 5) could be explained by low VlsE content in whole cell vaccine formulations, because in vitro VlsE expression depends on temperature and pH and our conditions were not optimal for VlsE^51^.

The p83 antigen is mainly recognized by the sera of patients with late borreliosis, and as a marker of the late phase^52,53^, it is also mentioned together with p58 and lipid antigens in the manuals of the kits used. It indicates hypothetically that alternative morphological forms could participate on generation of p83 antibodies in advanced infection and thus could be present at higher level at this stage of infection. However, lipid antigens and p58 were not recognized by any of the tested sera, so it can be assumed that a natural infection is necessary for the response to them. Our results also indicate an increased expression of OspC on *Borrelia* aggregates, although a study of others focused on the possible role of RpoN/RpoS alternative sigma factors pathways on *Borrelia* aggregation did not find differences in the transcript level compared to free spiral cells^32^.

In order to determine the influence of cell structures preservation on the immunogenicity of *Borrelia,* we next compared inactivated whole cell vaccines, tested above, with respective bacterial cell lysate vaccines, generated by bacteria sonication (Fig. 7). For all three morphological types, we found a higher immunogenicity of formaldehyde inactivated whole cell preparations, which preferentially induced antibodies of the IgG2a isotype, associated with the pro-inflammatory Th1 type of immune response. Parthasarathy and colleagues compared in vitro inflammatory reaction of cultured oligodendrocytes to heat inactivated and sonicated *Borrelia burgdorferi* and showed that both can induce inflammatory reaction consisting in the production of CCL2, IL-6, and CXCL8 with higher levels when exposed to heat killed *Borrelia*^54^. Subsequently Parthasarathy et al. compared the response of the nervous system explants of frontal cortex and dorsal root ganglion from rhesus monkeys brain to live and sonicated *Borrelia* focusing on CCL2, IL-6, and CXCL8 expression and found that both live and sonicated *Borrelia* could induce all three mediators in both brain region tissues^55^. Our results thus support the hypothesis that *Borrelia* can maintain pathogenic potential even in the non-viable forms leading to the persistence of inflammation untill complete clearance. According to our results, the preservation of the cell structure could affect the intensity of immune response (Fig. 7). On the other hand comparison of immunogenicity of tested morphological variants (aggregates, RB and spirals) did not show significant differences. Immunopathological inflammatory reaction can itself cause clinically manifest tissue damage, but it can also participate in triggering an autoimmune reaction, which is often discussed in connection with post-infectious complications of LD, mainly treatment-resistant Lyme arthritis^56,57^.

The exact mechanism of the possible induction of an autoimmune reaction by *Borrelia* infection is not yet known, but the mechanism of molecular mimicry has long been discussed^58–61^. However, in the more recent literature, do not relying on mimicry phenomenon, autoantibodies against a wide range of different autoantigens have been described in patients with LD or PTLDS. Specifically, it concerns antibodies against endothelial cell growth factor^62^, matrix metalloproteinase-10^63^, apolipoprotein B-100^64^, anti-ganglioside autoantibodies^65^, anti-phospholipid antibodies^66^, anti-nuclear and myositis-specific/associated autoantibodies^13,14^.

In order to detect possible cross-reactivity, we tested the sera of mice immunized with individual *Borrelia* morphotypes for the presence of antibodies reacting with human nuclear and myositis autoantigens using the immunoblot method. For all analyzed morphological type vaccines tested, we did not notice a positive or borderline reaction with any of the autoantigens included (Supplementary Fig. 7), and our results thus do not support the contribution of cross-reactivity to the formation of autoantibodies against human autoantigens. The absence of detectable autoantibodies induced by cross-reactivity during the vaccination experiments does not exclude the possibility that during long duration of LD in human they are elicited. Differences could be caused by short course of our murine immunization experiment, suboptimal inflammation induced by administered vaccines and species-linked differences in autoantigens structure and sensitivity to break autotolerance. Our results, in combination with the wide spectrum of described specificities of detected autoantibodies, indicate that in human LD other mechanisms, such as polyclonal activation, suppression of tolerance, antigen spreading or exposure of cryptic antigens during inflammatory process induced by *Borrelia* infection (and possibly maintained by persistent antigenic structures), are more likely involved in the induction of the autoimmune reaction than simple cross-reactivity.

Although our study based on the *in-vitro* induction of morphological changes of spirochetes has significant limitations for drawing clinical conclusions, the increased antigenicity of bacterial aggregates in patients after experiencing the late phase of infection, in combination with a higher ability of aggregates to induce the formation of antibodies against a marker of advanced infection, suggests the possible applicability of aggregates in the development of new LD diagnostics.

## Material and methods

### *Borrelia* cultivation and induction of different morphological forms

Spirochetes were grown in BSK-H Medium with L-Glutamine (BioConcept, Schwitzerland) supplemented with 6% rabbit serum suitable for *Borrelia* culture (Sigma-Aldrich, Germany) and Antibiotic mixture for *Borrelia* containing amphotericin B, phosphomycin and rifampicin (Himedia, India). The cultures were incubated at 33 °C. Bacteria from culture in the logarithmic growth phase were used as spiral forms. Round bodies were induced by the addition of 100 µg/ml ampicillin sodium salt (Merck, USA) for 48 h. Aggregates formation was induced by prolonged cultivation time (approx. three weeks). Before further processing, spiral forms and round bodies were washed three times with sterile PBS (centrifugation at 6000×*g*, 20 °C, 10 min). Repeated PBS washing with low-speed centrifugation was used to isolate aggregates from spiral cells in culture (5 cycles, 50×*g*, 20 °C, 10 min).

Change in morphology was monitored by fluorescence microscopy using carboxyfluorescein succinimidyl ester vital staining^67^. In the case of round bodies, the staining was performed before the induction of the morphological change.

The following strains were used in the experiment: *Borrelia burgdorferi* sensu stricto 35210, *Borrelia bissettii* CIP 109136, *Borrelia afzelii* (field isolate from *I. ricinus*, Czech Republic) and *Borrelia garinii* (field isolate from *I. ricinus*, Czech Republic). The *B. garinii* strain was chosen for the vaccination experiments due to its growth characteristics and good transition to alternative forms, in addition, it is one of the two dominant species in the territory of the Czech Republic.

### Characteristics of the studied patient groups and blood sampling

Patients were selected by physicians of the cooperating clinical departments based on clinical suspicion of early or late stage LD. Patients with positive laboratory evidence of antibodies against *Borrelia* in both IgM and IgG classes during primary diagnosis were included in the Early stage group, and patients with repeated positivity of only IgG antibodies with positivity of one or more of the markers of advanced infection—p58, p83 or lipid antigens—were included in the Late stage group. The control group represented the patients of the Department of Neurology of the University Hospital Olomouc examined negative for Lyme disease bacteria by PCR in cerebrospinal fluid and *Borrelia*-specific antibodies in the cerebrospinal fluid and serum in the course of differential diagnosis of neurological disorders. Patients with a negative result of all of *Borrelia*-specific tests with final diagnosis of demyelinating disorder of the multiple sclerosis type were included.

Blood samples were collected by trained medical staff from the Department of Allergology and Clinical Immunology, the Third Department of Internal Medicine-Nephrology, Rheumatology and Endocrinology, and the Department of Immunology of Olomouc University Hospital, Olomouc Region, or provided by the Laboratory of Medical Parasitology and Zoology of the Institute of Public Health Ostrava, Moravian-Silesian Region, Czech Republic. The samples were collected from patients with clinical suspicion of Lyme disease from 2019 to 2021.

The study was conducted according to the guidelines of the Declaration of Helsinki. The protocol of the study, including the informed consent of the patients, was approved by the Ethics Committee of the Olomouc University Hospital (reference number 102/18 of June 2018). Informed consent was obtained from all participants.

*Borrelia* antibodies tests for inclusion in groups were performed using the Anti-*Borrelia* EUROLINE-RN-AT IgM and IgG blot diagnostic kits (EUROIMMUN, Lübeck, Germany) with evaluation by a flatbed scanner and software EUROLineScan Software 3.4 (EUROIMMUN, Lübeck, Germany) in accordance with the manufacturer's instructions.

### Testing of human antibodies against individual morphological forms of *Borrelia*

Wells of ELISA plates (Multisorp) were coated with 100 μl of lysates of individual *Borrelia* morphological forms with a protein concentration of 10 μg/ml in carbonate buffer (100 mM bicarbonate/carbonate, pH 9.6) at 4 °C overnight. The plates were washed with PBS-T solution (PBS buffer containing 0.05% Tween, pH 7.4), and wells were blocked by blocking solution (5% dry skimmed milk in PBS-T). Human sera samples were applied at a 1:200 dilution in PBS-T supplemented with 1% of bovine serum albumin (BSA, Merck, USA). Samples were incubated at 4 °C overnight, wells were 3× washed with PBS-T and secondary antibody (Anti-Mouse IgG γ-chain specific—Peroxidase antibody produced in goat, Sigma-Aldrich, Germany) was applied at diluted 1:5000 in PBS-T with 1% BSA. After two hours of incubation at room temperature, wells were washed 4× with PBS-T, 2× with PBS and OPD tablet (Sigma-Aldrich Germany) in Phosphate-Citrate buffer (Sigma-Aldrich, Germany) was used to visualize the reaction. The reaction was stopped by 1 M sulphuric acid, and absorbance at 450 nm was measured using BioTek Synergy HTX multi-mode reader. The test with one morphotype lysate was always performed on the same ELISA panel for all three groups of subjects.

Reactivity against spirals snd aggregates lysate was determined also by western blotting. Briefly, standard protocol SDS-PAGE was used to separate the protein using Bio-Rad Protean II system with 12% gel and preparative comb. A sample with a protein content of 0.5 mg/ml was mixed with reducing loading buffer 1:1 and 100 μl was applied to one gel. Separated proteins were transferred to a PVDF membrane using a semi-dry transfer unit. The membrane was subsequently blocked with 5% dry skimmed milk in PBS-T, washed and incubated with individual sera in the group diluted 1:500 in PBS-T with 1% BSA using Mini-protean II multi screen apparatus (Bio-Rad, USA). After washing with PBS-T, the strips were incubated with Anti-Human IgG conjugate 10x (Euroimmun, Germany), diluted in PBS-T with 1% BSA. After further washing, the reaction was visualized by chromogenic substrate (Euroimmun, Germany).

### Immunization experiments

Whole cell vaccines were prepared by inactivating washed bacteria of individual morphological forms with 1% formaldehyde in PBS, pH 7.5. Incubation was carried out for 10 days at 4 °C. Subsequently, the bacteria were washed 5 times with PBS, each time with a five-minute incubation on a rotary mixer. The protein concentration in individual preparations was determined by the BCA method (Pierce™ BCA Protein Assay Kit, Thermo Fisher Scientific, USA). The bacterial suspension was then mixed with an aluminum adjuvant VAC 20 (SPI Pharma, France). The final vaccine contained 200 µg/ml protein and 20% aluminum hydroxide.

The lysates were prepared by resuspending the live bacteria in PBS with 0.1 mM EDTA and 0.5% phenoxyethanol followed by lysis with three freeze–thaw cycles and ten cycles of sonication on ice (10 s cycles). Vaccines were prepared from the lysate as in the case of whole cell antigen (200 μg/ml of protein, 20% of aluminum adjuvant).

All experiments were performed on 6- to 8-week old female BALB/c mice purchased from Anlab (Prague, Czech Republic). All animals were free of known pathogens and were kept in a climate-controlled environment and were provided with pellet food and water ad libitum. Each of the mice was vaccinated twice intradermally with 100 µl of vaccine (containing 20 µg of protein) with a two-week interval between doses. A group of four mice was immunized with each vaccine. Two weeks after the second dose, mice were euthanized under Ketamine/Xylazine anesthesia and blood samples were taken from which serum was isolated by centrifugation.

The Ethics Committee of the Faculty of Medicine and Dentistry (Palacky University, Olomouc, Czech Republic) and the Ministry of Education, Youth and Sports, Czech Republic authorized the immunization experiment on mice. All procedures were in accordance with ARRIVE guidelines and American Veterinary Medical Association Guidelines for the Euthanasia of Animals.

### Evaluation of the immunological response of vaccinated mice

The ELISA method was used to evaluate the levels of *Borrelia* specific IgG, IgG1 and IgG2a. Coating and blocking was performed in the same way as in the case of human antibody determination, but only the lysate of spiral forms was used. The tests were performed analogously to those used for human sera. The lysate of spiral forms was used for coating the ELISA plates at concentration 10 μg/ml. Mouse sera were applied in dilutions 1:3200 for total IgG and 1:500 for IgG subclasses. Detection of *Borrelia* specific Ig levels was done using anti-IgG (Sigma-Aldrich, Germany, dilution 1:5000), anti-IgG1 (BD, USA, dilution 1:1000), and anti-IgG2a (BD, USA, dilution 1:1000) HRP conjugates as a secondary antibodies.

Two human diagnostic sets were also employed to evaluate the antibody response against individual *Borrelia* antigens—a line blot set Anti-*Borrelia* EUROLINE-RN-AT (IgG)(Euroimmun, Geermany) based on purified recombinant antigens and Anti-*Borrelia* EUROLINE-WB (IgG) set (Euroimmun, Germany) based on electrophoretically separated antigen extracts of *B. afzelii* supplemented by a membrane chip with recombinant VlsE antigen. For use with mouse sera, the anti-human antibody conjugates included in the kits were replaced with Goat Anti-Mouse IgG Fc, AP-conjugated (Invitrogen, USA) diluted 1:2000. Except for this change, assays were performed according to the manufacturer's instructions using the buffers and substrate included in the kits. Both sets used enable a software densitometric evaluation of the intensity of the reaction with individual antigens after scanning the processed strip. All blot tests in our experiments were evaluated by EUROLineScan Software 3.4 (EUROIMMUN, Lubeck, Germany). An example of the evaluation of the strips can be found in the Supplementary Fig. S4. Using this software with the EUROLINE WB test, it is possible to identify and evaluate the reaction with the following antigens—p17 (DbpA), p19, p21, p25 (OspC), p30, p31 (OspA), p39 (BmpA), p83 and VlsE. EUROLINE-RN-AT then contains membrane chips with recombinant antigens p17 (DbpA), p19, p20, p21, p25 (OspC), p39 (BmpA), p41 (FlaB), p58, p83, three variants of VlsE (*B. burgdorferi* s.s., *B. afzelii* and *B. garinii*) and extracted lipid antigens of *B. burgdorferi* s.s. and *B. afzelii*. To construct the figures, images of individual strips were cut from the output of the evaluation software and compiled in Microsoft PowerPoint software.

Reactivity of mouse sera with aggregate lysates was also tested. Determination of total specific IgG was performed using ELISA protocol described previously. Western blot analysis with aggregates lysates was performed following the procedure analogous as for human sera using preparative combs to prepare gels. The procedure differed in that the strips cutted from membrane and each strip was individually incubated with pools of individual sera in the group diluted 1:100 in PBS-T with 1% BSA using plastic 10 channels incubation tray (TestLine, Czech Republic). After washing with PBS-T, the strips were incubated with Goat Anti-Mouse IgG Fc, Alkaline Phosphatase (AP) Conjugate (Invitrogen, USA), diluted 1:5,000 in PBS-T with 1% BSA. Sera from individual mice were not tested separately due to insufficient sample volume.

### Determination of autoantibodies in the mouse sera

An immunoblotting method was used to determine the potential cross-reactivity of *Borrelia* antigens with human autoantigens. We used diagnostic kits EUROLINE Autoimmune Inflammatory Myopathies 16 Ag (IgG) containing the following antigens: Mi-2a, Mi-2b, TIF1g, MDA5, NXP2, SAE1, Ku, PM-Scl100, PM-Scl75, Jo-1, SRP, PL-7, PL-12, EJ, OJ, Ro-52 and EUROLINE ANA profile 3 plus DFS70 (IgG) kit containing antigens nRNP/Sm, Sm, SS-A, SS-B, Scl-70, Jo-1, dsDNA, nucleosomes, histones, ribosomal P-protein, AMA M2, Ro-52, PM-Scl, CENP B, PCNA, and DFS70 (both EUROIMMUN, Luebeck, Germany). As Jo-1 and Ro-52 antigens are included in both sets, a result was obtained for a total of 30 autoantigens. For use with mouse sera, as in the case of the *Borrelia* antibodies tests, the secondary antibody contained in the kit was replaced by an anti-mouse IgG AP-conjugate antibody.

### Statistical analysis

All the graph, calculation, and statistical analyses were performed using GraphPad Prism software version 8.01 for Windows (GraphPad Software, San Diego, California, USA). Data were analyzed using one-way analysis of variance (ANOVA) followed by post hoc tests (Tukey’s test). The Kruskal–Wallis test followed by Dunn’s post hoc test was used for evaluation of densitometric data due to the content of null values among the results. The p-values less than 0.05 were considered as statistically significant.

### Supplementary Information

Supplementary Figures.

## Data Availability

The datasets used and/or analyzed during the current study are available from the corresponding author on reasonable request.
