# Supplementary material for: Antigenicity and immunogenicity of different morphological forms of Borrelia burgdorferi sensu lato spirochetes
Source: Sci Rep. 2024 Feb 18;14:4014. doi: 10.1038/s41598-024-54505-y (PMC10874929; doi:10.1038/s41598-024-54505-y)
Supplement: Supplementary file 1 — Supplementary Figures. [file 41598_2024_54505_MOESM1_ESM.pdf]

Supplementary Information

Antigenicity and immunogenicity of different morphological forms of *Borrelia burgdorferi* sensu lato spirochetes.

Kristyna Sloupenska, Barbora Koubkova, Pavel Horak, Jana Dolezilkova, Beata Hutyrova, Mojmir Racansky, Martina Miklusova, Jan Mares, Milan Raska, Michal Krupka

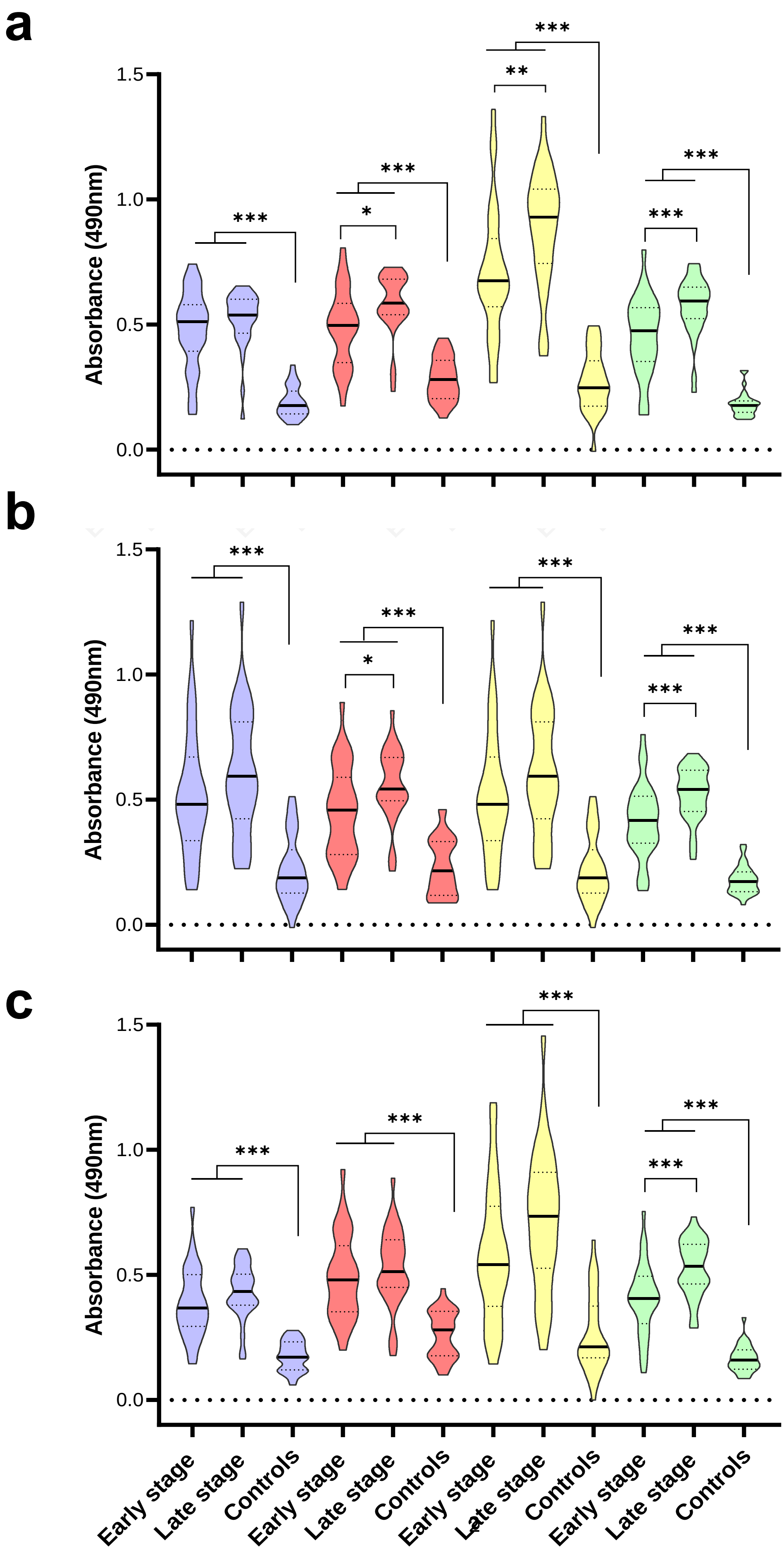
a
b
c
Absorbance (490nm)
1.5
1.0
0.5
0.0
***
**
*
Early stage
Late stage
Controls
Early stage
Late stage
Controls
Early stage
Late stage
Controls
Early stage
Late stage
Controls

Supplementary Figure S1: Reactivity of LD patients and control subjects sera with lysates of individual morphotypes of different species of Bbsl. The sera of subjects seropositive for markers of early stage of LD (n=30), late stage of LD (n=30) and seronegative control subjects (n=30) were tested. *Borrelia*-specific IgG antibodies were determined by the ELISA method with panels coated with lysates of individual morphotypes. Results are shown as absorbance of individual wells at a wavelength of 490 nm at a 100x sample dilution. Blue – *B. burgdorferi* s.s., red – *B. afzelii*, yellow – *B. garinii*, green – *B. bissettii*. Panels were coated with lysate of: a-aggregates, b-round bodies and c-spiral forms. Full line – average of group values, dotted lines - quartiles of group values, *p<0.05; **p<0.01; ***p<0.001.

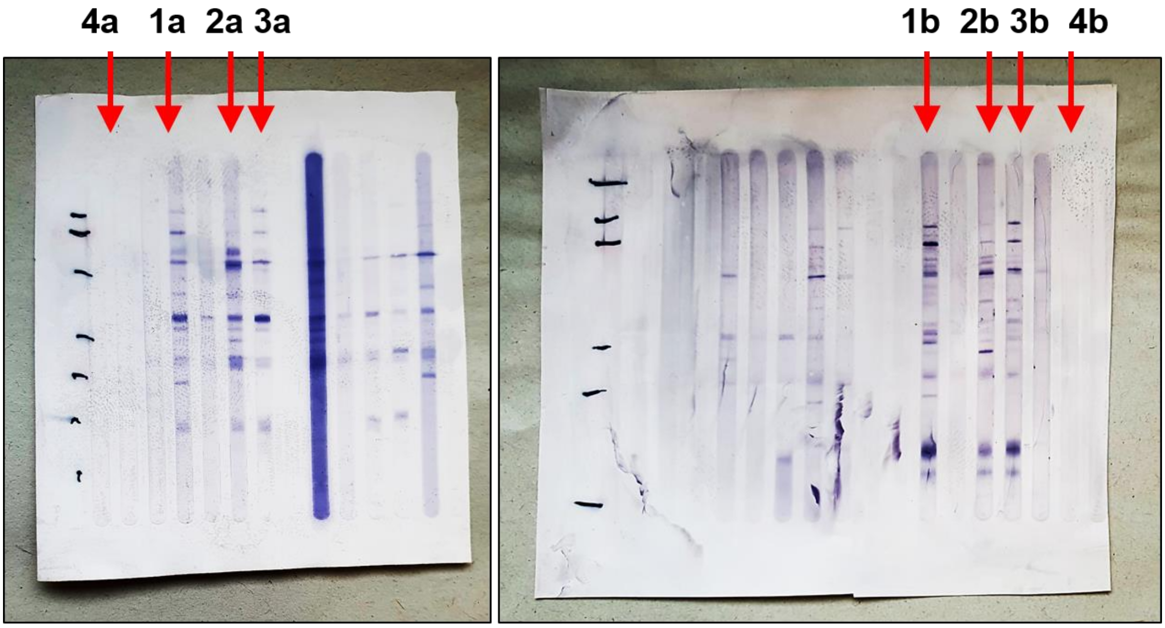

Supplementary Figure S2: Immunoblot analysis of the reactivity of patients sera with lysates of spiral forms and aggregates of *B. garinii*. Multiscreen blot membranes used as source for Figure 3.

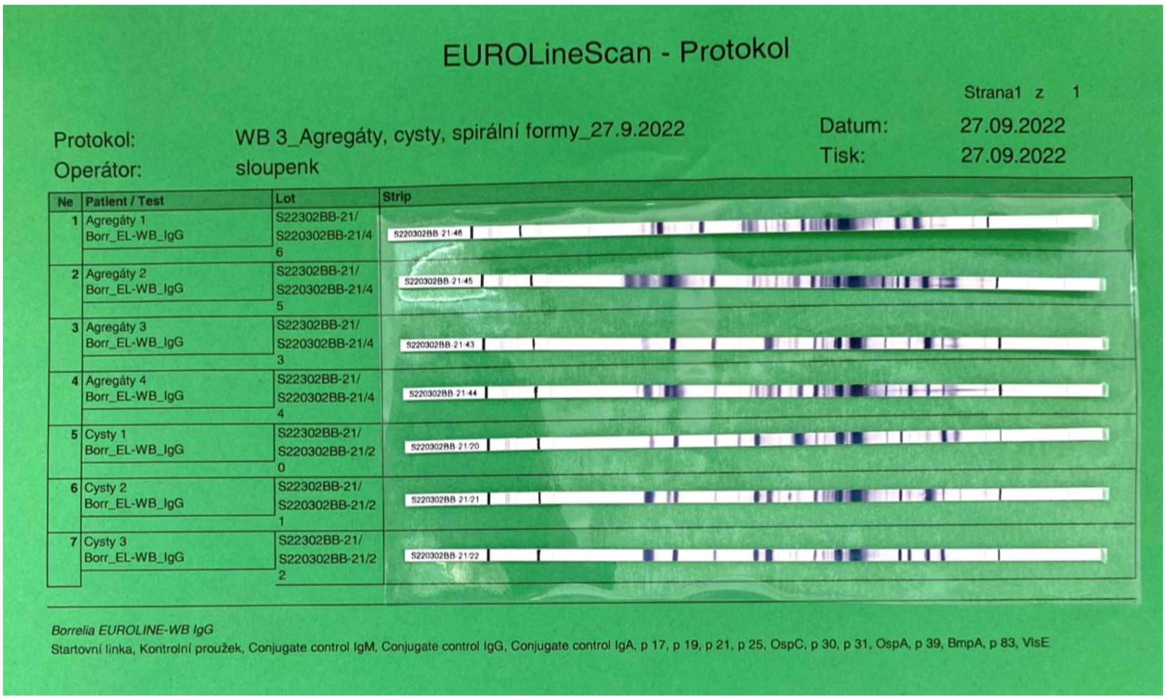

**Pacient ID:**	Agregáty 1	**Test:**	Borrelia EUROLINE-WB IgG
**Vytvořit:**	27.09.2022	**Jamka:**	1
**Výsledky z:**			

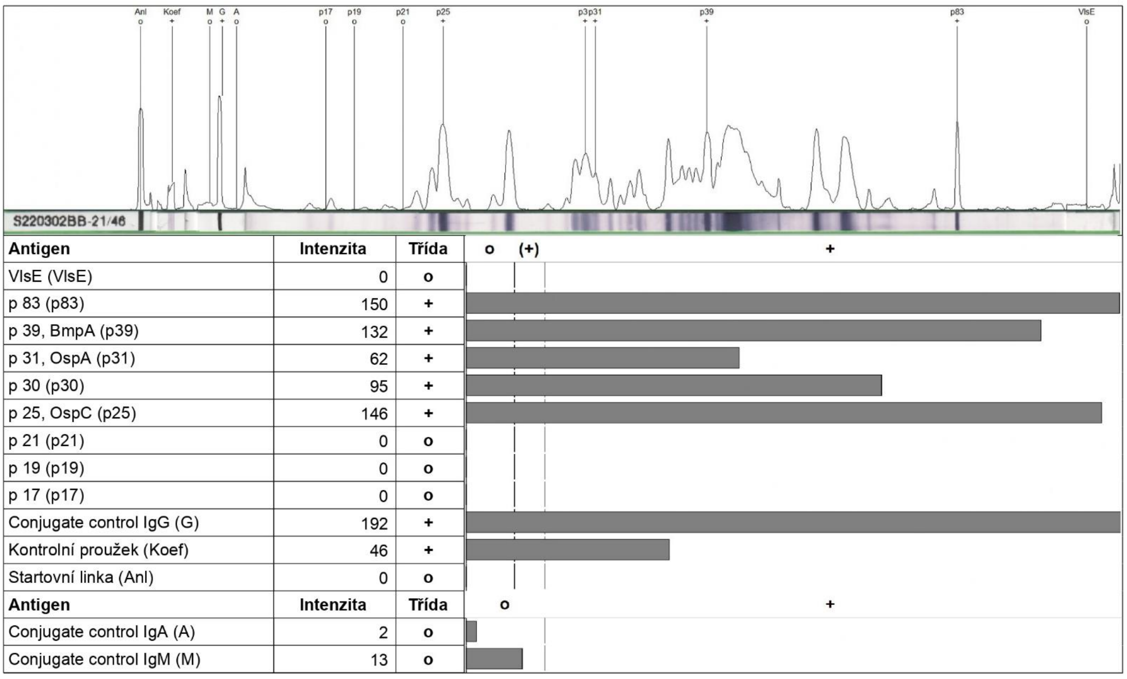

Antigen	Intenzita	Třída
VlsE (VlsE)	0	o
p 83 (p83)	150	+
p 39, BmpA (p39)	132	+
p 31, OspA (p31)	62	+
p 30 (p30)	95	+
p 25, OspC (p25)	146	+
p 21 (p21)	0	o
p 19 (p19)	0	o
p 17 (p17)	0	o
Conjugate control IgG (G)	192	+
Kontrolní proužek (Koef)	46	+
Startovní linka (Anl)	0	o

Antigen	Intenzita	Třída
Conjugate control IgA (A)	2	o
Conjugate control IgM (M)	13	o

Test	Výsledek
Borrelia EUROLINE-WB IgG	pozitivní

Supplementary Figure S3: Western blot analysis of sera of mice immunized with inactivated individual morphological types of *B. garinii*. Example images of blot strips adhered to the evaluation protocol (top) and individual software densitometric analyze of a single strip (bottom).

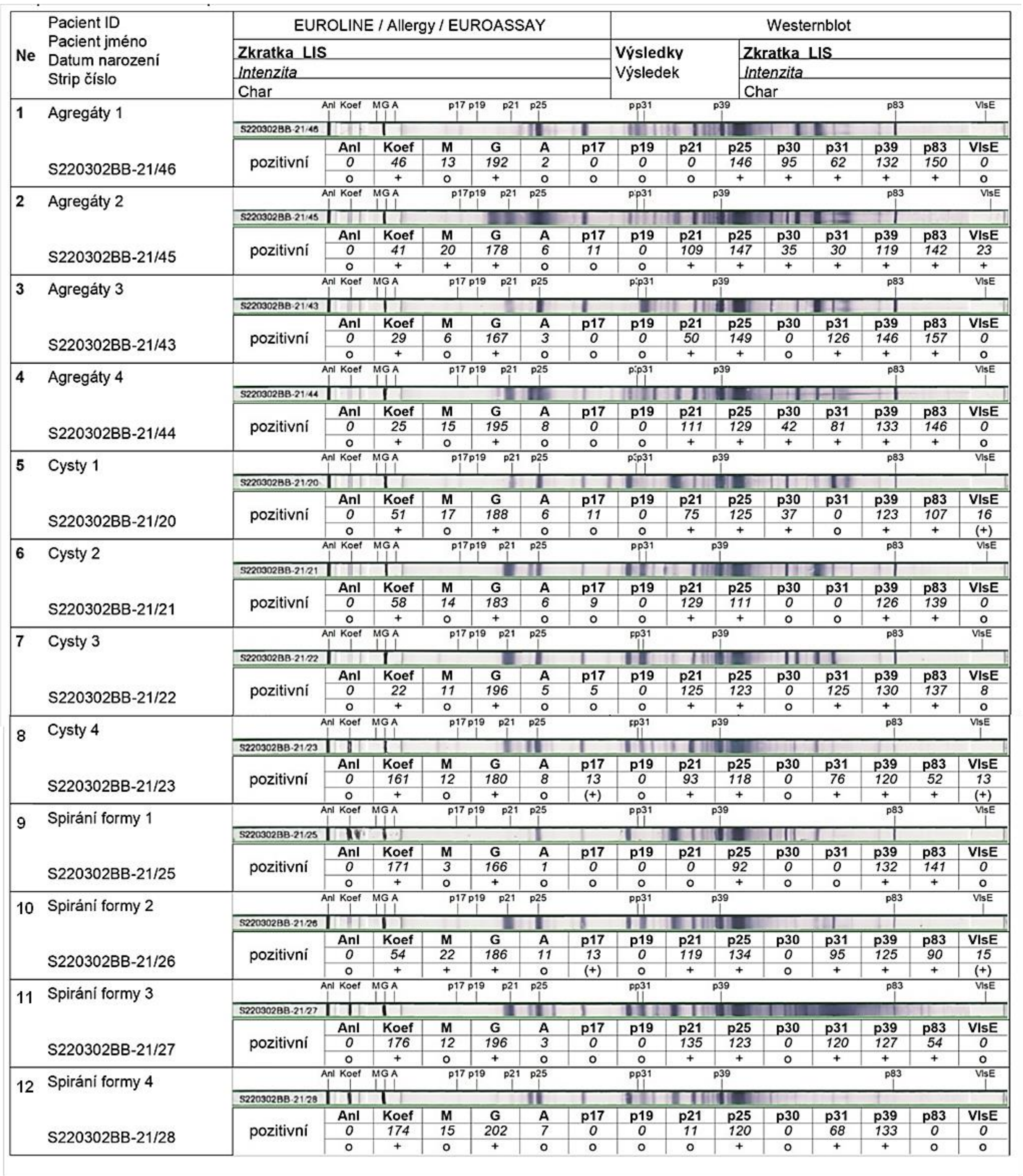

Ne	Pacient ID / Pacient jméno / Datum narození / Strip číslo	Výsledek	Řádek	Anl	Koef	M	G	A	p17	p19	p21	p25	p30	p31	p39	p83	VlsE
1	Agregáty 1 / S220302BB-21/46	pozitivní	Intenzita	0	46	13	192	2	0	0	0	146	95	62	132	150	0
			Char	o	+	o	+	o	o	o	o	+	+	+	+	+	o
2	Agregáty 2 / S220302BB-21/45	pozitivní	Intenzita	0	41	20	178	6	11	0	109	147	35	30	119	142	23
			Char	o	+	+	+	o	o	o	+	+	+	+	+	+	+
3	Agregáty 3 / S220302BB-21/43	pozitivní	Intenzita	0	29	6	167	3	0	0	50	149	0	126	146	157	0
			Char	o	+	o	+	o	o	o	+	+	o	+	+	+	o
4	Agregáty 4 / S220302BB-21/44	pozitivní	Intenzita	0	25	15	195	8	0	0	111	129	42	81	133	146	0
			Char	o	+	o	+	o	o	o	+	+	+	+	+	+	o
5	Cysty 1 / S220302BB-21/20	pozitivní	Intenzita	0	51	17	188	6	11	0	75	125	37	0	123	107	16
			Char	o	+	o	+	o	o	o	+	+	+	o	+	+	(+)
6	Cysty 2 / S220302BB-21/21	pozitivní	Intenzita	0	58	14	183	6	9	0	129	111	0	0	126	139	0
			Char	o	+	o	+	o	o	o	+	+	o	o	+	+	o
7	Cysty 3 / S220302BB-21/22	pozitivní	Intenzita	0	22	11	196	5	5	0	125	123	0	125	130	137	8
			Char	o	+	o	+	o	o	o	+	+	o	+	+	+	o
8	Cysty 4 / S220302BB-21/23	pozitivní	Intenzita	0	161	12	180	8	13	0	93	118	0	76	120	52	13
			Char	o	+	o	+	o	(+)	o	+	+	o	+	+	+	(+)
9	Spirání formy 1 / S220302BB-21/25	pozitivní	Intenzita	0	171	3	166	1	0	0	0	92	0	0	132	141	0
			Char	o	+	o	+	o	o	o	o	+	o	o	+	+	o
10	Spirání formy 2 / S220302BB-21/26	pozitivní	Intenzita	0	54	22	186	11	13	0	119	134	0	95	125	90	15
			Char	o	+	+	+	o	(+)	o	+	+	o	+	+	+	(+)
11	Spirání formy 3 / S220302BB-21/27	pozitivní	Intenzita	0	176	12	196	3	0	0	135	123	0	120	127	54	0
			Char	o	+	o	+	o	o	o	+	+	o	+	+	+	o
12	Spirání formy 4 / S220302BB-21/28	pozitivní	Intenzita	0	174	15	202	7	0	0	11	120	0	68	133	0	0
			Char	o	+	o	+	o	o	o	o	+	o	+	+	o	o

Supplementary Figure S4: Western blot analysis of sera of mice immunized with inactivated individual morphological types of *B. garinii* – software evaluated blot strips, source data for Figure 4. Each lane represents the serum of one vaccinated mouse. 1 – 4 – mice immunized with aggregates, 5 – 8 – mice immunized with round bodies, 9 – 12 – mice immunized with spiral forms. IgG control bands (G) were artificially created by a laboratory marker, because when using the originally diagnostic human test with mouse serum, they are not formed, but the blots without them are not able to be evaluated by the original software. The results themselves are not affected by this technical intervention.

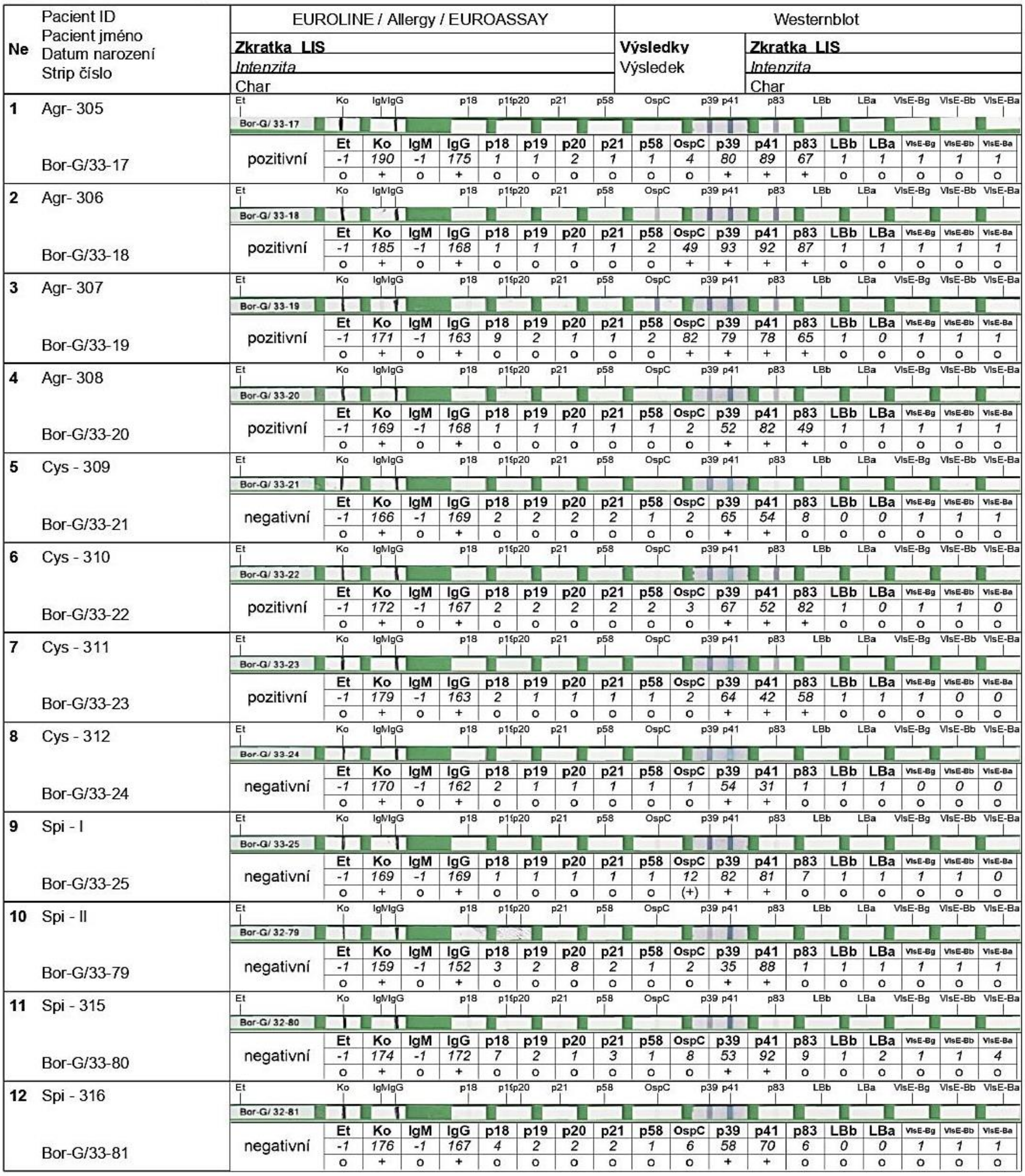

Ne	Pacient ID / Pacient jméno / Datum narození / Strip číslo	Strip	Výsledky / Výsledek	Zkratka LIS	Et	Ko	IgM	IgG	p18	p19	p20	p21	p58	OspC	p39	p41	p83	LBb	LBa	VlsE-Bg	VlsE-Bb	VlsE-Ba
1	Agr- 305 / Bor-G/33-17	Bor-G/ 33-17	pozitivní	Intenzita	-1	190	-1	175	1	1	2	1	1	4	80	89	67	1	1	1	1	1
				Char	o	+	o	+	o	o	o	o	o	o	+	+	+	o	o	o	o	o
2	Agr- 306 / Bor-G/33-18	Bor-G/ 33-18	pozitivní	Intenzita	-1	185	-1	168	1	1	1	1	2	49	93	92	87	1	1	1	1	1
				Char	o	+	o	+	o	o	o	o	o	+	+	+	+	o	o	o	o	o
3	Agr- 307 / Bor-G/33-19	Bor-G/ 33-19	pozitivní	Intenzita	-1	171	-1	163	9	2	1	1	2	82	79	78	65	1	0	1	1	1
				Char	o	+	o	+	o	o	o	o	o	+	+	+	+	o	o	o	o	o
4	Agr- 308 / Bor-G/33-20	Bor-G/ 33-20	pozitivní	Intenzita	-1	169	-1	168	1	1	1	1	1	2	52	82	49	1	1	1	1	1
				Char	o	+	o	+	o	o	o	o	o	o	+	+	+	o	o	o	o	o
5	Cys - 309 / Bor-G/33-21	Bor-G/ 33-21	negativní	Intenzita	-1	166	-1	169	2	2	2	2	1	2	65	54	8	0	0	1	1	1
				Char	o	+	o	+	o	o	o	o	o	o	+	+	o	o	o	o	o	o
6	Cys - 310 / Bor-G/33-22	Bor-G/ 33-22	pozitivní	Intenzita	-1	172	-1	167	2	2	2	2	2	3	67	52	82	1	0	1	1	0
				Char	o	+	o	+	o	o	o	o	o	o	+	+	+	o	o	o	o	o
7	Cys - 311 / Bor-G/33-23	Bor-G/ 33-23	pozitivní	Intenzita	-1	179	-1	163	2	1	1	1	1	2	64	42	58	1	1	1	0	0
				Char	o	+	o	+	o	o	o	o	o	o	+	+	+	o	o	o	o	o
8	Cys - 312 / Bor-G/33-24	Bor-G/ 33-24	negativní	Intenzita	-1	170	-1	162	2	1	1	1	1	1	54	31	1	1	1	0	0	0
				Char	o	+	o	+	o	o	o	o	o	o	+	+	o	o	o	o	o	o
9	Spi - I / Bor-G/33-25	Bor-G/ 33-25	negativní	Intenzita	-1	169	-1	169	1	1	1	1	1	12	82	81	7	1	1	1	1	0
				Char	o	+	o	+	o	o	o	o	o	(+)	+	+	o	o	o	o	o	o
10	Spi - II / Bor-G/33-79	Bor-G/ 32-79	negativní	Intenzita	-1	159	-1	152	3	2	8	2	1	2	35	88	1	1	1	1	1	1
				Char	o	+	o	+	o	o	o	o	o	o	+	+	o	o	o	o	o	o
11	Spi - 315 / Bor-G/33-80	Bor-G/ 32-80	negativní	Intenzita	-1	174	-1	172	7	2	1	3	1	8	53	92	9	1	2	1	1	4
				Char	o	+	o	+	o	o	o	o	o	o	+	+	o	o	o	o	o	o
12	Spi - 316 / Bor-G/33-81	Bor-G/ 32-81	negativní	Intenzita	-1	176	-1	167	4	2	2	2	1	6	58	70	6	0	0	1	1	1
				Char	o	+	o	+	o	o	o	o	o	o	+	+	o	o	o	o	o	o

Supplementary Figure S5: Line blot analysis of sera of mice immunized with inactivated individual morphological types of *B. garinii* – software evaluated blot strips, source data for Figure 5. Each lane represents the serum of one vaccinated mouse. 1 – 4 – mice immunized with aggregates, 5 – 8 - mice immunized with round bodies, 9 – 12 – mice immunized with spiral forms. For technical reasons, control bands were additionally created similar to the previous image, which did not affect the results of the rest of the blot.

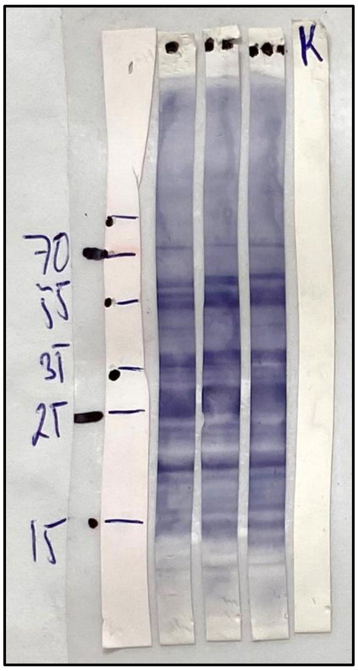

Supplementary Figure S6: Specific reactivity of serum IgG from mice immunized with inactivated whole cell vaccines generated from individual morphological types of *B. garinii.* Original scan of blot membrane strips used as source for Figure 6. Each strip was incubated separately with pool of specific sera of mice from one immunization group.

A

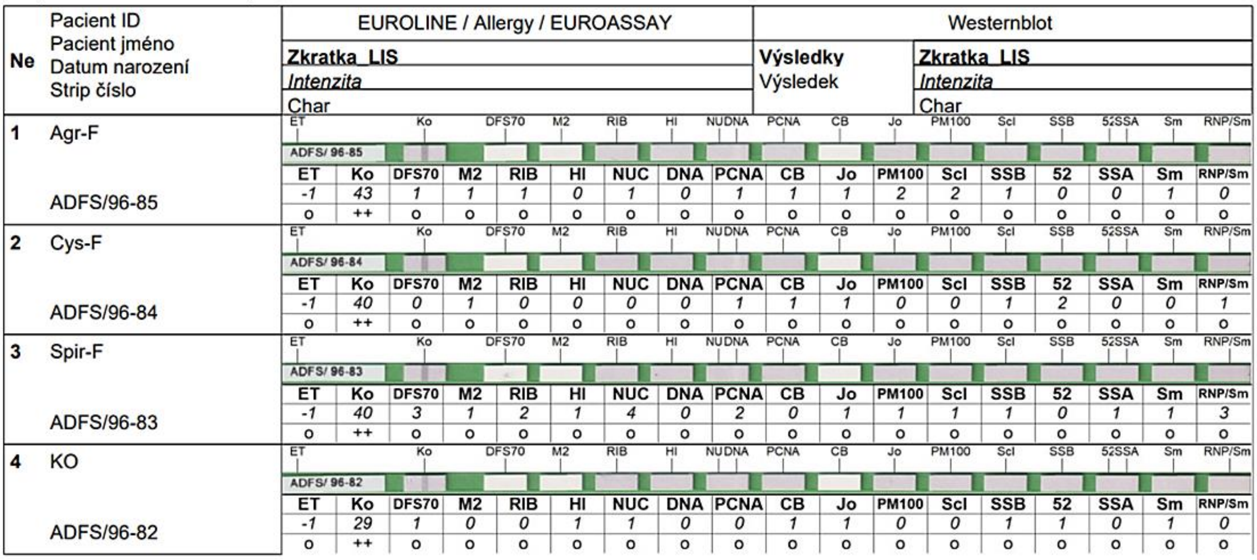

Ne	Pacient ID / Pacient jméno / Datum narození / Strip číslo		ET	Ko	DFS70	M2	RIB	HI	NUC	DNA	PCNA	CB	Jo	PM100	Scl	SSB	52	SSA	Sm	RNP/Sm
1	Agr-F ADFS/96-85	Intenzita	-1	43	1	1	1	0	1	0	1	1	1	2	2	1	0	0	1	0
		Char	o	++	o	o	o	o	o	o	o	o	o	o	o	o	o	o	o	o
2	Cys-F ADFS/96-84	Intenzita	-1	40	0	1	0	0	0	0	1	1	1	0	0	1	2	0	0	1
		Char	o	++	o	o	o	o	o	o	o	o	o	o	o	o	o	o	o	o
3	Spir-F ADFS/96-83	Intenzita	-1	40	3	1	2	1	4	0	2	0	1	1	1	1	0	1	1	3
		Char	o	++	o	o	o	o	o	o	o	o	o	o	o	o	o	o	o	o
4	KO ADFS/96-82	Intenzita	-1	29	1	0	0	1	1	0	0	1	1	0	0	1	1	0	1	0
		Char	o	++	o	o	o	o	o	o	o	o	o	o	o	o	o	o	o	o

B

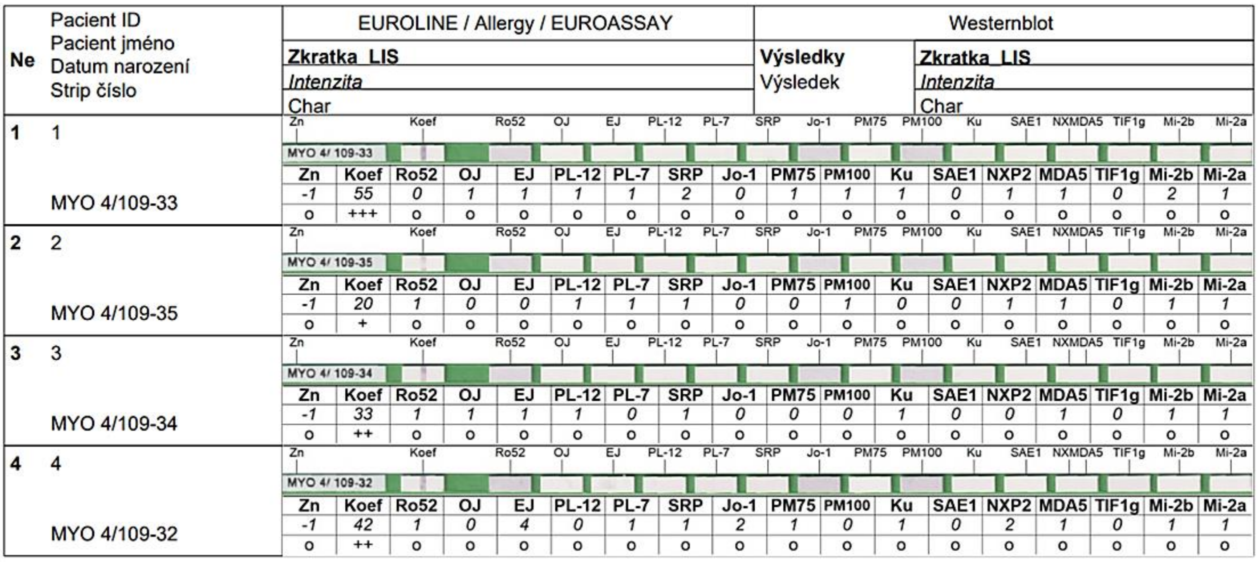

Ne	Pacient ID / Pacient jméno / Datum narození / Strip číslo		Zn	Koef	Ro52	OJ	EJ	PL-12	PL-7	SRP	Jo-1	PM75	PM100	Ku	SAE1	NXP2	MDA5	TIF1g	Mi-2b	Mi-2a
1	1 MYO 4/109-33	Intenzita	-1	55	0	1	1	1	1	2	0	1	1	1	0	1	1	0	2	1
		Char	o	+++	o	o	o	o	o	o	o	o	o	o	o	o	o	o	o	o
2	2 MYO 4/109-35	Intenzita	-1	20	1	0	0	1	1	1	0	0	1	0	0	1	1	0	1	1
		Char	o	+	o	o	o	o	o	o	o	o	o	o	o	o	o	o	o	o
3	3 MYO 4/109-34	Intenzita	-1	33	1	1	1	1	0	1	0	0	0	1	0	0	1	0	1	1
		Char	o	++	o	o	o	o	o	o	o	o	o	o	o	o	o	o	o	o
4	4 MYO 4/109-32	Intenzita	-1	42	1	0	4	0	1	1	2	1	0	1	0	2	1	0	1	1
		Char	o	++	o	o	o	o	o	o	o	o	o	o	o	o	o	o	o	o

Suplementary Figure S7: Results of determination of antibodies against human autoantigens in mice vaccinated with different formaldehyde inactivated morphotypes of *B. garinii*. A – antinuclear antibodies kit ANA Profile 3 plus DFS70, B – myositis antibodies kit Autoimmune inflamatory Myopathies 16 Ag (both Euroimmun, Germany). Test strips were incubated with pooled sera of mice vaccinated with: 1 – aggregates, 2 – round bodies, 3 – spiral forms. 4 – pool of naive mice sera.

Autoantibodies against the following autoantigens were tested:

Antinuclear autoantibodies: nRNP/Sm, Sm, SS-A/Ro, Ro-52, SS-B/La, Scl-70 (topoisomerase I), PM-Scl, Jo-1 (histidyl-tRNA synthetase), CENP B (centromere protein B), PCNA (proliferating cell nuclear antigen), dsDNA, nucleosomes, histones, ribosomal P-proteins, AMA M2, DFS70 (dense fine speckled, 70kDa molecular weight protein).

Myositis autoantibodies: Mi-2a, Mi-2b, TIF1g (transcriptional intermediary factor 1) MDA5 (melanoma differentiation-associated protein 5), NXP2 (nuclear matrix protein 2), SAE1 (small ubiquitin-like modifier 1-activating enzyme), Ku, PM-Scl100, PM-Scl75, Jo-1 (histidyl-tRNA synthetase), SRP (signal recocgnition particle), PL-7 (threonyl-tRNA sythetase), PL-12 (alanyl-tRNA synthetase), EJ (glycyl-tRNA synthetase), OJ (isoleucyl-tRNA synthetase), Ro-52.
